# Bacteria-Derived Extracellular Vesicles in Urine as a Novel Biomarker for Gastric Cancer: Integration of Liquid Biopsy and Metagenome Analysis

**DOI:** 10.3390/cancers13184687

**Published:** 2021-09-18

**Authors:** Jae-Yong Park, Chil-Sung Kang, Ho-Chan Seo, Jin-Chul Shin, Sung-Min Kym, Young-Soo Park, Tae-Seop Shin, Jae-Gyu Kim, Yoon-Keun Kim

**Affiliations:** 1 Department of Internal Medicine, Chung-Ang University College of Medicine, Seoul 06973, Korea; jay0park@cau.ac.kr; 2 Institute of MD Healthcare Inc., Seoul 03923, Korea; cskang@mdhc.kr (C.-S.K.); hcseo@mdhc.kr (H.-C.S.); jcsin@mdhc.kr (J.-C.S.); tsshin@mdhc.kr (T.-S.S.); 3 Division of Infectious Diseases, Department of Internal Medicine, Sejong Chungnam National University Hospital, Sejong 30099, Korea; smkimkor@cnu.ac.kr; 4 Department of Internal Medicine, Seoul National University Bundang Hospital, Seongnam 13620, Korea; dkree@snubh.org

**Keywords:** extracellular vesicles, gastric cancer, liquid biopsy, biomarker, microbiome, 16S rRNA amplicon, metagenomics

## Abstract

**Simple Summary:**

Gastric cancer shows an improved prognosis when diagnosed in its early stage. However, non-invasive diagnostic markers for gastric cancer known to date have poor clinical efficacies. Many studies have shown that gastric cancer patients have distinct microbial changes compared to normal subjects. In the present study, we performed metagenome analysis using body fluid samples (gastric juice, blood, and urine) to investigate the distinct microbial composition using bacteria-derived EVs from gastric cancer patients. We could build diagnostic prediction models for gastric cancer with the metagenomic data and analyzed the accuracy of models. Although further validation is required to apply these findings to real clinical practice yet, our study showed the possibility of gastric cancer diagnosis with the integration of liquid biopsy and metagenome analysis.

**Abstract:**

Early detection is crucial for improving the prognosis of gastric cancer, but there are no non-invasive markers for the early diagnosis of gastric cancer in real clinical settings. Recently, bacteria-derived extracellular vesicles (EVs) emerged as new biomarker resources. We aimed to evaluate the microbial composition in gastric cancer using bacteria-derived EVs and to build a diagnostic prediction model for gastric cancer with the metagenome data. Stool, urine, and serum samples were prospectively collected from 453 subjects (gastric cancer, 181; control, 272). EV portions were extracted from the samples for metagenome analysis. Differences in microbial diversity and composition were analyzed with 16S rRNA gene profiling, using the next-generation sequencing method. Biomarkers were selected using logistic regression models based on relative abundances at the genus level. The microbial composition of healthy groups and gastric cancer patient groups was significantly different in all sample types. The compositional differences of various bacteria, based on relative abundances, were identified at the genus level. Among the diagnostic prediction models for gastric cancer, the urine-based model showed the highest performance when compared to that of stool or serum. We suggest that bacteria-derived EVs in urine can be used as novel metagenomic markers for the non-invasive diagnosis of gastric cancer by integrating the liquid biopsy method and metagenome analysis.

## 1. Introduction

Although the incidence of gastric cancer has been steadily decreasing worldwide, it still remains one of the most common and fatal causes of cancer death in the world [1]. The 5-year survival rate of gastric cancer is particularly low in the advanced stage [2]. However, the survival rate is much higher in countries where the majority of gastric cancers are newly diagnosed in early stages. This finding is evident in Korea and Japan, where a national screening program for gastric cancer is provided [3,4].

Endoscopic examination with pathologic confirmation is the primary diagnostic modality for gastric cancer. Although relatively safe, endoscopy is an invasive procedure and can cause serious complications occasionally. It can also be a burden in terms of cost. Liquid biopsy is considered the most promising area for cancer diagnosis in that it can be an alternative to traditional tissue biopsy methods, taking advantage of the recent development of next-generation sequencing (NGS) technology [5]. The monitoring system using liquid biopsy was initially studied based on cell-free DNA in blood, but it has recently developed by using body fluids such as stool, urine, or saliva. Several studies have been conducted or are now underway for serological diagnosis of gastric cancer [6]. However, there are no non-invasive markers with significant accuracy in the early diagnosis of gastric cancer yet, which can be used in real clinical settings.

*Helicobacter pylori* infection is a well-known risk factor for gastric cancer development. Recently, the rapid development of microbiome research has focused much attention on the possibility that microbiome other than *H. pylori* may be involved in the gastric carcinogenesis process [7,8]. The human gastrointestinal tract is the most complex ecosystem well known. The commensal microorganisms in it affect not only the immune system but also numerous physiological and metabolic processes in the human body, suggesting their role in the pathogenesis of various diseases [9,10]. Many studies are underway to establish the relationship between gastric cancer and microbiomes [7,8,11]. Gut microbe-derived extracellular vesicles (EVs) are emerging as novel proof of the relationship between commensal bacteria and host health conditions [12,13]. The EVs can act as intercellular communication mediators carrying various cargoes, including signaling molecules and transcription factors. Many studies have shown that EVs are associated with various immune responses in humans, causing inflammation or inhibiting reactions [14,15]. However, in the context of gastric carcinogenesis, few studies have been reported focusing on microbiome-derived EVs. Studies linking the EVs to early diagnosis of gastric cancer are even more scarce.

In this context, we conducted a metagenome analysis using microbiome-derived EVs in stool, urine, and serum samples from a large number of prospective cohorts of gastric cancer patients and healthy controls. After identifying the potential microbial biomarkers, we developed diagnostic prediction models for gastric cancer with various types of liquid biopsy samples and validated the diagnostic performance of each model.

## 2. Materials and Methods

### 2.1. Subjects and Sample Collection

In total, 272 healthy people (159 males and 113 females) and 181 gastric cancer patients (122 males and 59 females) were enrolled from Haewoondae Baek Hospital (Busan, Korea), Chung-Ang University Hospital (Seoul, Korea), and Seoul National University Bundang Hospital (Seongnam, Gyeonggi-do, Korea) between December 2016 and December 2019. Among the patient’s medical records, medical history, age, sex, endoscopic diagnosis, and pathologic results were reviewed. The inclusion criteria for the gastric cancer group were patients newly diagnosed with gastric cancer who did not undergo endoscopic or surgical resection or chemotherapy yet. The healthy control group included those without evidence of dysplasia or gastric cancer on the endoscopic examination. Patients were excluded if they had a previous history of gastrointestinal surgery, were pregnant, or were taking antibiotics, probiotics, or acid-suppressing drugs within the previous 3 months, as these conditions can temporarily alter the gut microbial composition. The minimum duration of drug cessation was determined by referring to previous literature [16,17,18,19]. This study protocol was approved by the Institutional Review Board of Haewoondae Baek Hospital (IRB No. 129792-2015-064), Chung-Ang University Hospital (IRB No. 1772-001-290), and Seoul National University Bundang Hospital (IRB No. B-1708/412-301). Stool, serum, and urine samples were collected from the subjects for metagenomics analyses. All participants ate a regular Korean diet a day before sampling and did not smoke or drink alcohol. The regular Korean diet is characterized by high levels of whole grains, vegetables, and low levels of animal-derived foods and saturated fat, particularly in contrast to the Western-style diet [20]. The stool sample was collected from the center of the stool, and placed in a sterilized container, and stored at −20 °C. For serum collection, we drew 3 mL of blood from each subject in an SST tube. The tube was then centrifuged (3000× *g*, 15 min, 4 °C) immediately after collection, and the serum was extracted. The supernatant was stored in Eppendorf tube 1 mL each at −20 °C. For urine, 40 mL of midstream urine was collected at a clean urine container and transferred to a conical tube, which was kept frozen at −20 °C.

### 2.2. Bacterial and EV Isolation and DNA Extraction from Clinical Samples

Bacteria EVs were isolated from the urine and serum of individuals following the procedure described previously [21,22]. Briefly, each urine sample was centrifuged at 10,000× *g* for 10 min at 4 °C. The supernatant was taken and passed through a 0.22 μm membrane filter to eliminate foreign particles and then quantified based on protein concentration. For serum, after mixing 100 μL of serum and 900 μL PBS, it was centrifuged at 10,000× *g* for 10 min at 4 °C to eliminate other components. The supernatant was taken and passed through a 0.22 μm membrane filter to eliminate foreign particles, and it was quantified based on protein concentration. The stool sample was mixed with PBS for dilution in a 1:10 ratio (1 g:10 mL) and maintained at 4 °C for 24 h. After dilution, the sample was centrifuged (10,000× *g*, 10 min, 4 °C) to separate the bacteria portion and the EV portion. The rest of the procedure was carried out in the same way as urine and serum.

Bacterial DNA extraction from prepared EVs was performed as described previously [23,24]. Briefly, isolated EVs (1 μg by protein, each sample) were boiled at 100 °C for 40 min, centrifuged at 13,000× *g* for 30 min, and the supernatants were collected. Collected samples were then subjected to bacterial DNA extraction using a DNA extraction kit (PowerSoil DNA Isolation Kit, MO BIO, Carlsbad, CA, USA) following the manufacturer’s instructions. Isolated DNA was quantified by using the QIAxpert system (QIAGEN, Hilden, Germany). In the case of stool, DNA was extracted by dividing the pellet (including bacteria portion) and the supernatant (including EV portion) to compare each.

### 2.3. PCR Amplification, Library Construction, and Sequencing of 16S rRNA Gene Variable Regions

To perform microbiome analysis, 16S rRNA gene amplicon metagenome analysis was conducted. Prepared bacterial DNA was used for PCR amplification of the V3-V4 hypervariable regions of the 16S ribosomal RNA genes using the primer set of 16S_V3_F (5ʹ-TCGTCGGCAGCGTCAGATGTGTATAAGAGACAGCCTACGGGNGGCWGCAG-3ʹ) and 16S_V4_R (5ʹ-GTCTCGTGGGCTCGGAGATGTGTATAAGAGACAGGACTACHVGGGTATCTAATCC-3ʹ). The PCR products were used for the construction of 16S rRNA gene libraries following the MiSeq System guidelines (Illumina Inc., San Diego, CA, USA). The 16S rRNA gene libraries for each sample were quantified using QIAxpert (QIAGEN, Germany), pooled at the equimolar ratio, and used for pyrosequencing with the MiSeq System (Illumina Inc., San Diego, CA, USA) according to the manufacturer’s recommendations.

### 2.4. Analysis of Bacterial Composition in the Microbiome

Paired-end reads were trimmed by cutadapt (ver. 1.1.6). The resulting FASTQ files containing paired-end reads were merged using CASPER and then quality filtered by Phred (Q) score. After merging, any reads under 350 bp or over 550 bp were also discarded. Next, a reference-based chimera detection step was performed using VSEARCH against the SILVA gold database. The sequence reads were clustered into operational taxonomic units (OTUs) using the de novo clustering algorithm, and the threshold was 97% sequence similarity. Finally, OTUs were classified using UCLUST under default parameters with SILVA 132 database.

### 2.5. Diagnostic Prediction Models for Gastric Cancer

The selection of biomarkers for the diagnostic model was based on relative abundances at the genus level. Candidates for bacterial biomarkers were chosen based on two criteria: the statistically significant difference (*p* < 0.05) between control and cancer subjects, the average relative abundance of 0.1% or more for each group. The whole data sets were randomly divided into training sets and test set in a ratio of 8:2. The prediction model was constructed using logistic regression models for each sample type, using the training set. The biomarkers to be used in the model were selected through a stepwise method by eliminating unnecessary variables, and Akaike’s information criterion (AIC) was used as a criterion for selection variables in the prediction model. After the diagnostic prediction models were built, the performance was evaluated on the test set for validation.

### 2.6. Statistical Analysis

To clarify the species abundancy between healthy control and gastric cancer group, alpha diversity of the variance within each clinical sample was assessed using the alpha diversity test in the phyloseq package in R for the total observed OTUs, richness estimates Chao1, and the Shannon and Simpson diversity indices. In order to avoid the alpha diversity bias, we rarified with the minimum read value for each sample. Dimension reduction was conducted to assess the beta diversity between clinical samples based on the Bray–Curtis dissimilarity using principal coordinate analysis (PCoA) and multiple dimension scale (MDS) in the stats package in R. Permutational multivariate analysis of variance (PERMANOVA) was used to validate either the centroid or the spread of each sample are different between the groups. The significant difference between the control group and the gastric cancer group was determined using a *t*-test, and *p*-values were adjusted using the Benjamini–Hochberg method to reduce the false discovery rates. Receiver operating characteristic (ROC) curves of gastric cancer diagnostic prediction models were developed through stepwise selection of significantly altered genera. The performance of the models was evaluated by assessing the area under the ROC curve (AUC), sensitivity, specificity, and accuracy. *p* value < 0.05 was considered statistically significant.

## 3. Results

### 3.1. Clinical Characteristics of Subjects

A total of 453 subjects were registered, including 181 gastric cancer patients and 272 healthy controls. Age and sex were matched between each sample group. A total of 813 samples from enrolled subjects were used for analysis. There was no statistical difference in sex and age between the gastric cancer group and control group in all four sample types: ST-Bac (bacteria portion extracted by using centrifuged pellet in stool), ST-EV (EV portion extracted by using centrifuged supernatant in stool), urine, and serum (Table 1).

### 3.2. Comparison of Alpha and Beta Diversity between Healthy Controls and Gastric Cancer Patients

**Figure 1:**
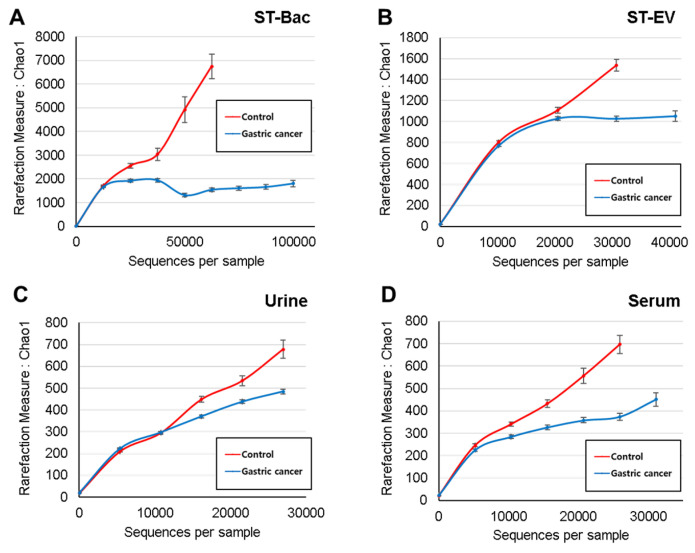
Comparison of alpha diversity between healthy controls and gastric cancer patients in 4 sample types. Estimated species richness (Chao1 measure) is demonstrated for the two groups (**A**) in stool bacteria (ST-Bac) isolated from stool pellet, (**B**) in stool-derived extracellular vesicle (ST-EV) isolated from stool supernatant, (**C**) in urine, and (**D**) in serum.

Read numbers of 16S rRNA amplicons and OTU counts derived from the NGS results are shown in Table 2. In stool samples, alpha diversity did not show significant differences between the two groups for all the diversity indices (Figure S1A,B). In urine and serum samples, Simpson index and Chao1 index showed significant differences between the two groups, respectively (Figure S1C,D). However, the differences in alpha diversity were generally not obvious. To evaluate the alpha diversity that includes all samples, a rarefaction curve analysis was performed with the Chao1 index to calculate species abundance per sequence. The slope of the rarefaction curve was steeper in the healthy control group than in the gastric cancer group in all sample types, indicating higher alpha diversity in the control group than in the gastric cancer group (Figure 1A–D).

**Figure 2:**
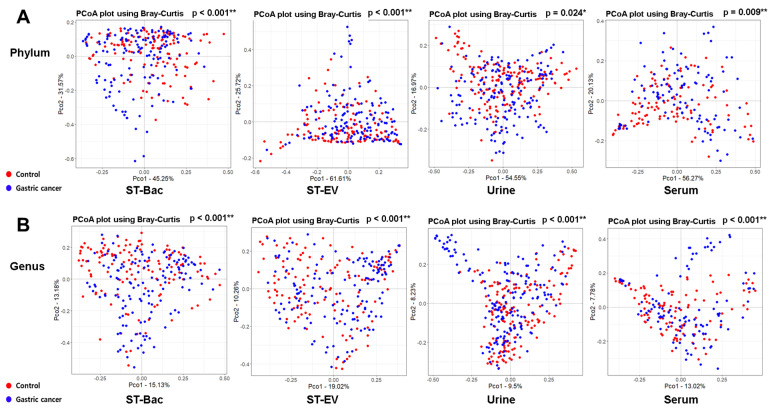
Comparison of beta diversity in phylum and genus levels between healthy controls and gastric cancer patients in 4 sample types. PCoA results based on Bray–Curtis similarity for beta diversity of bacteria are shown (**A**) in phylum level and (**B**) in genus level.

At the phylum level, the beta diversity showed significant differences between the control group and the gastric cancer group in all sample types (Figure 2A). At the genus level, these differences were repeatedly confirmed, with even higher statistical significances (Figure 2B). Although we could not detect a significant reduction in microbial diversity in gastric cancer, the microbial composition in the gastric cancer group was significantly different from that in healthy controls.

### 3.3. Relative Abundance Differences between Healthy Controls and Gastric Cancer Patients

**Figure 3:**
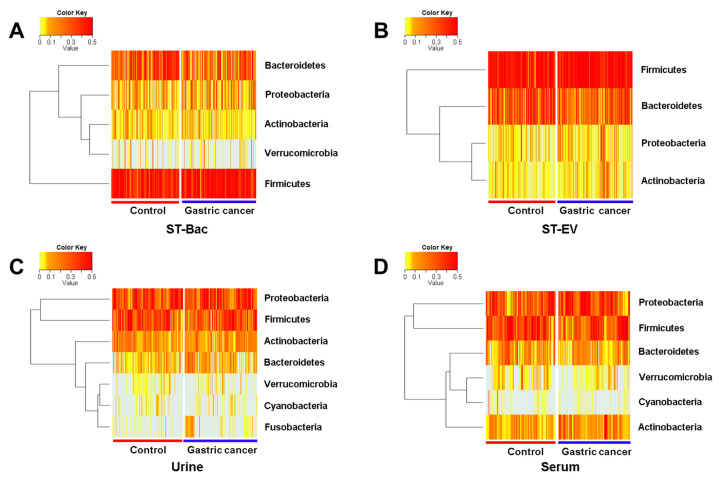
Relative abundance differences in phylum level between healthy controls and gastric cancer patients in 4 sample types. Heatmaps representing the relative abundances of microbiome between the two groups (**A**) in stool bacteria (ST-Bac) isolated from stool pellet, (**B**) in stool-derived extracellular vesicle (ST-EV) isolated from stool supernatant, (**C**) in urine, and (**D**) in serum.

We compared the relative abundances of microbiome between the control group and the gastric cancer group to identify the taxa that were differentially represented in the two groups of subjects. In the comparison of phylum levels, heatmaps using ST-Bac samples showed that *Bacteroidetes* were reduced, while *Firmicutes* were increased in gastric cancer (Figure 3A and Figure S2A). In ST-EV samples, *Bacteroidetes* were reduced, while *Actinobacteria* were increased in gastric cancer (Figure 3B and Figure S2B). In urine samples, *Bacteroidetes* and *Fusobacteria* were both increased in gastric cancer, differently from ST-Bac and ST-EV samples (Figure 3C and Figure S2C). In serum samples, *Verrucomicrobia* were reduced, while *Actinobacteria* were increased in gastric cancer (Figure 3D and Figure S2D).

**Figure 4:**
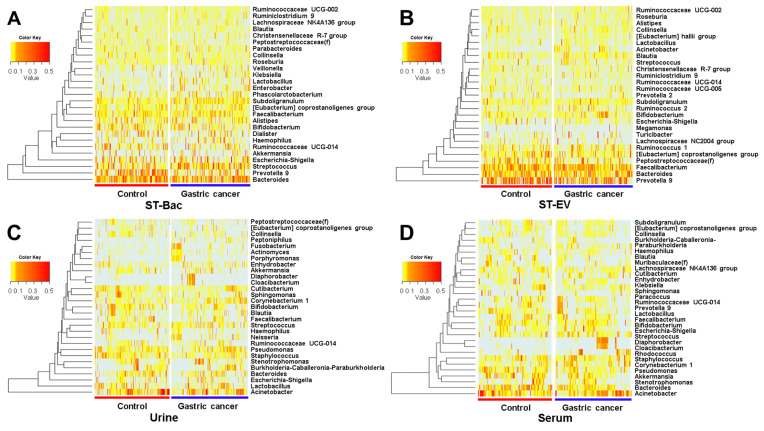
Relative abundance differences in genus level between healthy controls and gastric cancer patients in 4 sample types. Heatmaps representing the relative abundances of microbiome between the two groups (**A**) in stool bacteria (ST-Bac) isolated from stool pellet, (**B**) in stool-derived extracellular vesicle (ST-EV) isolated from stool supernatant, (**C**) in urine, and (**D**) in serum.

**Figure 5:**
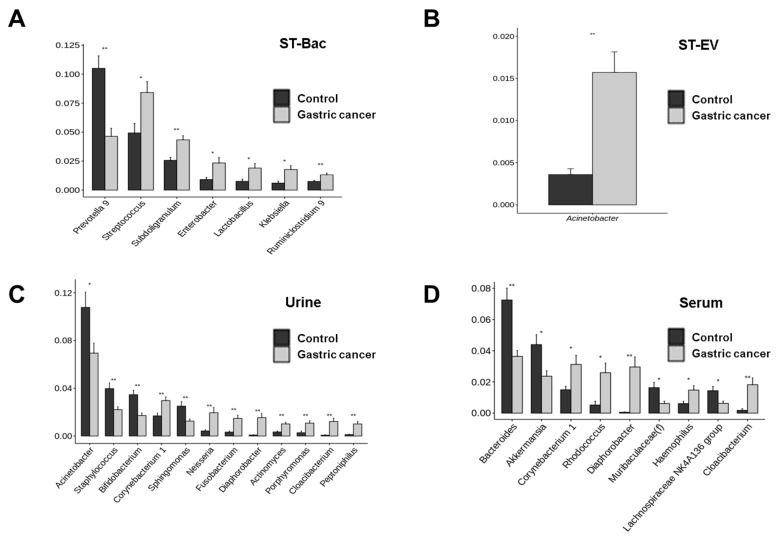
Compositional differences in genus level between healthy controls and gastric cancer patients in 4 sample types. The bar plots highlight the average relative abundance of individual key taxa between the two groups (**A**) in stool bacteria (ST-Bac) isolated from stool pellet, (**B**) in stool-derived extracellular vesicle (ST-EV) isolated from stool supernatant, (**C**) in urine, and (**D**) in serum. *, *p* < 0.05; **, *p* < 0.01.

When we compared the relative abundances in genus level, more bacteria became candidates showing the differences between the control group and gastric cancer group. In ST-Bac samples, *Prevotella 9* was decreased, while *Streptococcus*, *Subdoligranulum, Enterobacter, Lactobacillus*, *Klebsiella*, *Ruminiclostridium 9* were increased in gastric cancer (Figure 4A and Figure 5A). In ST-EV samples, *Acinetobacter* was increased in gastric cancer (Figure 4B and Figure 5B). In urine samples, the largest number of bacterial candidates were detected, revealing that *Acinetobacter*, *Stayphylococcus*, *Bifidobacterium*, and *Sphingomonas* were decreased, while *Corynebacterium 1*, *Neisseria*, *Fusobacterium, Diaphorobacter, Actinomyces, Porphyromonas, Cloacibacterium,* and *Peptoniphilus* were increased in gastric cancer (Figure 4C and Figure 5C). In serum samples, *Bacteroides*, *Akkermansia*, *Muribaculaceae(f)*, and *Lachnospiraceae NK4A136* were decreased, while *Corynebacterium 1, Rhodococcus*, *Diaphorobacter*, *Haemophilus*, and *Cloacibacterium* were increased in gastric cancer (Figure 4D and Figure 5D).

### 3.4. Comparison of Diagnostic Prediction Models for Gastric Cancer between Healthy Controls and Gastric Cancer Patients

**Figure 6:**
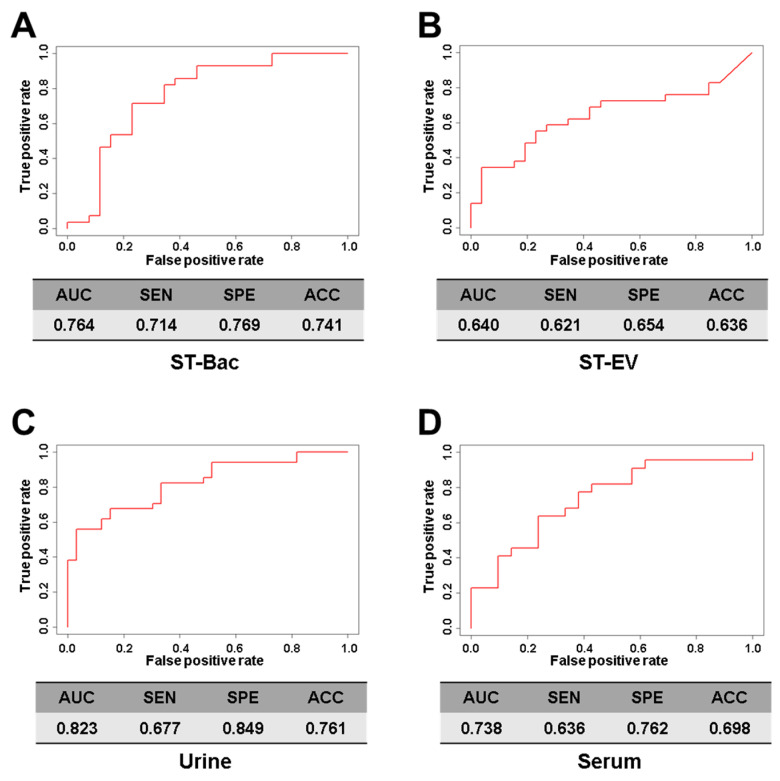
Comparison of gastric cancer diagnostic prediction models between healthy controls and gastric cancer patients in 4 sample types. ROC curves of gastric cancer diagnostic prediction models were developed through stepwise selection of significantly altered genera. Models were validated by performance evaluation in the test set by assessing the area under the curve (AUC), sensitivity, specificity, and accuracy. Performance indices of each model are demonstrated: (**A**) stool bacteria (ST-Bac) isolated from stool pellet, (**B**) stool-derived extracellular vesicle (ST-EV) isolated from stool supernatant, (**C**) urine, and (**D**) serum. SEN, sensitivity; SPE, specificity; ACC, accuracy.

To further define useful biomarkers from metagenomic biomarkers, optimal models were built using biomarkers to distinguish between gastric cancer group and control group using logistic regression analysis (Figure 6A–D). Selected variables included in the prediction models according to each sample type were as follows: ST-Bac-based model, *Klebsiella, Subdoligranulum, Prevotella 9, Streptococcus, Ruminiclostridium 9;* stool EV-based model, *Acinetobacter*; urine-based model, *Peptoniphilus, Diaphorobacter, Neisseria, Staphylococcus, Bifidobacterium, Corynebacterium 1, Actinomyces, Acinetobacter, Sphingomonas*; serum-based model, *Diaphorobacter, Bacteroides, Corynebacterium 1, Rhodococcus, Cloacibacterium, Haemophilus, Muribaculaceae(f), Akkermansia*.

As a result, the model using the urine samples showed the highest AUC value of 0.823 (Figure 6C). Although the sensitivity was rather low (67.7%) in this model, the specificity (84.9%) and accuracy (76.1%) were high when compared to other models. The ST-Bac-based model showed an AUC score of 0.764, lower than the urine-based model, but their sensitivity (71.4%), specificity (76.9%), and accuracy (74.1%) were generally high (Figure 6A). The ST-EV-based model and serum-based model revealed lower AUC values and accuracies than the two models mentioned above (Figure 6B,D).

To further analyze the performance of prediction models according to disease stages, we divided the gastric cancer group into early gastric cancer (EGC) and advanced gastric cancer (AGC) groups. The performance of the prediction model was higher in the AGC group than in the EGC group in all sample types except for ST-Bac Figure S3A–H). For EGC, the ST-Bac prediction model showed the highest AUC value of 0.830.

## 4. Discussion

We performed microbiome profiling of gastric cancer using various types of samples through analyzing the bacteria-derived EVs to find out diagnostic biomarkers for gastric cancer in correlation with gut microbes. There were significant differences in microbial composition between the gastric cancer group and the control group in all of the sample types. Diagnostic prediction models for gastric cancer were generated based on this information on metagenomic biomarkers, and the model using the urine samples showed the highest performance for the diagnosis of gastric cancer.

In this study, although there seemed to be a trend for reduced alpha diversity in gastric cancer, further analysis with various indices showed that the differences in microbial diversity between the two groups were not consistent among various sample types. This could also be indirectly inferred from the finding that the OTU values of the gastric cancer group and control group showed high standard deviations, which means that the number of microbial taxa in the two groups overlapped each other to some extent. This finding is different from previous reports, which suggest that reduced microbial diversity is often a characteristic feature of diseased status [25,26]. Several studies showed dysbiotic cancer-associated microbiome in gastric cancer, implying the role of microbial dysbiosis in gastric carcinogenesis [8,25]. This discrepancy may be due to several reasons. We used samples from extragastric area that reflect systemic circulation in this study, whereas samples obtained from the stomach (gastric juice, gastric mucosa, etc.) were mainly used in previous studies. Indirect analysis of microbiome using bacteria-derived EVs rather than direct identification of microbiome might also be a possible explanation. However, a comparison of beta diversity between the gastric cancer group and the control group revealed that microbial composition in the gastric cancer group was significantly different from that in healthy controls, which was evident with all four types of samples.

The composition of microbiota was further investigated with a relative abundance of taxa. The five most dominant bacterial phyla in stool were *Bacteroidetes*, *Proteobacteria*, *Actinobacteria*, *Verrucomicrobia*, and *Firmicutes*, which are consistent with previous literature [27,28]. EVs from these phyla were also highly abundant in urine and serum samples. This finding implies that bacteria-derived EVs in systemic circulation can roughly reflect the microbial composition of gastrointestinal microbes. Interestingly, *Proteobacteria* were dominant in urine and serum EVs, while *Firmicutes* and *Bacteriodetes* were the most abundant in stool samples at the phylum level. *Firmicutes* and *Bacteriodetes* generally comprise more than 90% of the human gut microbiome, according to previous studies [28], similar to the results from our study. In addition, *Firmicutes* were increased in stool, while EVs from *Firmicutes* did not show a significant change in serum in gastric cancer. The relative abundances of *Bacteroidetes* in stool and urine EVs also showed conflicting results. The difference in microbial composition was even more evident at the genus level. *Acinetobacter* (phylum *Proteobacteria*) were the most dominant in urine and serum EVs identically, whereas *Prevotella* and *Bacteroides* (phylum *Bacteriodetes*) were the most dominant in stool samples. Representative strains showing changes in gastric cancer were largely different between stool and urine/serum samples. Moreover, the gross pattern of the microbiome from urine and serum samples was similar, which can easily be inferred from the fact that urine is basically filtrated from blood during systemic circulation. This result shows that the microbial composition directly identified from the bacteria present in the gut lumen differs from the composition of bacteria indirectly inferred from the EVs, which are absorbed through the gut mucosa into the systemic circulation. It is recently recognized that bacteria secrete EVs, which are intercellular communicasomes between the host and commensal microbes, that can act as biologically active metabolites or as mediators of host-microbiome interaction [15,29,30]. Considering this, it can be inferred that there is a significant difference between the microbiome simply existing in the gastrointestinal tract and the core microbiome that are deeply involved in host-microbiome interaction, actually affecting the health of the host. In fact, gastric juice and feces contain lots of dead strains and strains with little clinical significance [31,32]. This might partly explain why the microbial diversity between the gastric cancer group and the control group was not consistent among various sample types. Although reduced microbial diversity in the gastrointestinal tract is often a characteristic feature of diseased status, this does not necessarily mean that the bacteria related to systemic circulation should also be less diverse in exactly the same manner. We do not yet fully understand which bacteria play major roles by host-microbiome interaction in certain diseases and how they systemically influence the disease pathogenesis. In fact, blood was known as a sterile specimen except for sepsis, but recent studies have shown that 16S rRNA gene-targeted NGS is possible with normal human blood, suggesting the presence of bacteria-derived EVs in blood. It is known that there is a difference in the EV composition in serum and plasma, and each of them has its inherent advantage according to the type of EVs and purpose of EV analysis [33,34,35]. Especially in terms of platelet richness in the plasma, there are reports that platelet-rich plasma (PRP) has some antibacterial effect [36,37]. Since 16s rRNA NGS analysis is based on bacteria, the platelet-poor plasma would be preferred over PRP in metagenome analysis using bacteria-derived EVs if plasma is used instead of serum. In brief, when performing microbiome analysis from the perspective of systemic effect on the host, it should be noted that circulating EVs found in blood or urine can have a greater meaning than analyzing samples directly obtained from the gastrointestinal tract.

We tried to explore the possibility of a prediction model that can be used for the early diagnosis of gastric cancer by using the microbiome data derived through EV analysis. Currently, some Asian countries, such as Korea and Japan, where the incidence of gastric cancer is very high, have been operating national cancer screening programs aiming to detect gastric cancer early. These programs increased the early detection of gastric cancer and lowered the mortality rate [3]. However, considering the socioeconomic costs of mass screening for a large number of people and the discomfort or complications from invasive procedures such as endoscopy, other new non-invasive diagnostic methods need to be developed. With the results of microbiome analysis, we investigate the possibility of liquid biopsy using diluted stool, urine, and serum.

In this study, prediction models were established for each sample type to distinguish gastric cancer patients from normal controls, and they were validated to evaluate the performance. The AUC value was higher than 0.7 in urine- and ST-Bac-based models, which is good performance according to the evaluation criteria (0.9–1.0 = excellent, 0.8–0.9 = very good, 0.7–0.8 = good, 0.6–0.7= sufficient, 0.5–0.6 = bad, <0.5). = not useful) [38], and the prediction model with urine showed the highest AUC value. However, although the specificity (84.9%) of the urine-based model was high, the sensitivity (67.7%) of this model was lower compared to that of the ST-Bac-based model (71.4%). When EGC and AGC groups were separately analyzed, although the ST-Bac-based prediction model showed the highest AUC value of 0.830 for EGC, the performances of prediction models were generally higher in the AGC group than in the EGC group except for ST-Bac. This indicates that the performances of prediction models differ by cancer stage and sample type. Accurate prediction of EGC seemed more difficult than AGC as one might easily expect, considering that the microbial changes would be more prominent in a more advanced state of the disease. In order to evaluate the suitability as a diagnostic test, it is necessary to consider various factors such as sensitivity, disease prevalence, cost-effectiveness, and convenience of sample collection. Especially, an ideal screening method should show adequate sensitivity and specificity, and there is typically a trade-off between these two performance metrics. High sensitivity is especially important in this situation, as a low value can lead to increased false-negative results, which means missing more cases with the disease of interest. Although the specificity of the urine-based model in our study was about 85%, the low sensitivity of the model prevents it from being a suitable screening method as it stands. However, despite the relatively low sensitivity of the urine-based model, it should also be considered that urine is much easier to collect than stool samples. Recent studies have also shown that the bacteria-derived EVs in urine samples contain characteristic features in allergic airway disease with a significant correlation with total IgE and eosinophil count, which further supports the possible role of microbial EVs in the implication of microbiota in the diseased state [39,40]. To further investigate the effect of *H. pylori* on the performance of the prognostic models, we tried to use stool samples to detect *H. pylori* first. However, *H. pylori* was detected in ST-Bac samples in only 13.6% of the cases. Furthermore, among the *H. pylori*-positive gastric cancer samples, the relative abundance of *H. pylori* was lower than 0.1% in all but one case. These findings made us assume that the validity of the analysis according to *H. pylori* status might be quite limited, especially when the detection rate and relative abundance of *H. pylori* are low. It is reasonable to investigate the gastric microbiome as a whole, the members of which influence each other and change the adjacent environment. Therefore, we decided to assess the diagnostic ability of prediction models using the whole integrated metagenome data, including *H. pylori*, as a comprehensive biomarker.

This study has some limitations. First, we did not perform the microbiome analysis according to *H. pylori* status. As *H. pylori* is the single most important pathogen for the gastric carcinogenesis process, this might have influenced the interpretation of study results. As the control subjects were recruited from health check-up programs, detailed histological information on the degree of mucosal atrophy was not available. Hypoacidity due to atrophic gastritis could alter the gut microbial composition, which is important in regions with high *H. pylori* prevalence, such as Korea. Therefore, the findings of the present study do not necessarily separate gastric cancer from atrophic pangastritis without cancer. Nevertheless, we showed a possibility of discriminating between gastric cancer patients and the general population without gastric cancer, using metagenomic data from a relatively large number of subjects. Further biomarker studies are needed for the detection of gastric cancer, and mucosal atrophy and *H. pylori* infection should be considered. Second, the performances of diagnostic prediction models were suboptimal. The urine-based model, which had the highest AUC value, showed rather low sensitivity. The results of our study further need to be verified through external validation.

## 5. Conclusions

In this study, we have shown that bacteria-derived EVs in systemic circulation can be used for demonstrating the changes in microbial composition in gastric cancer. Bacteria-derived EVs in urine harbors a potential as a new diagnostic biomarker for gastric cancer, suggesting that EVs might be a new standard substance for cancer diagnosis. Although it would be difficult to directly apply the results from this study to real clinical practice yet due to the suboptimal sensitivity and specificity of the diagnostic prediction models, these findings still have great significance in that they showed the possibility of integrating a liquid biopsy method with metagenome analysis for gastric cancer diagnosis.

## Figures and Tables

**Table 1:** Basal clinical characteristics of each sample group.

Sample Type	Age & Sex	Control	Gastric Cancer	*p*-Value
ST-Bac	Age (mean ± SD) Sex (M:F)	63.6 ± 8.3 127 (93:34)	63.6 ± 9.5 140 (95:45)	0.9815 0.4088
ST-EV	Age (mean ± SD) Sex (M:F)	63.6 ± 8.3 127 (93:34)	63.6 ± 9.5 141 (96:45)	0.9853 0.4307
Urine	Age (mean ± SD) Sex (M:F)	63.5 ± 9.8 164 (114:50)	63.8 ± 9.8 168 (114:54)	0.8207 0.8362
Serum	Age (mean ± SD) Sex (M:F)	62.3 ± 9.4 105 (74:31)	63.7 ± 10.3 108 (73:35)	0.2891 0.7590

SD, standard deviation; M, male; F, female.

**Table 2:** Read numbers of 16S rRNA amplicons and OTU counts from the NGS results.

Sample Type	Group	Read Count			OTU		
		Mean	Median	SE	Mean	Median	SE
ST-Bac	Control Gastric cancer	18,352.6 19,987.9	16,243.0 16,138.5	±1062.7 ±1420.5	939.4 890.4	810.0 763.5	±64.5 ±46.6
ST-EV	Control Gastric cancer	19,262.0 22,555.8	18,378.0 19,753.0	±961.6 ±1227.5	550.7 556.9	453.0 510.0	±32.7 ±22.7
Urine	Control Gastric cancer	12,766.7 12,968.7	12,412.5 9282.0	±598.8 ±787.5	176.6 167.4	140.5 141.5	±11.6 ±8.6
Serum	Control Gastric cancer	11,340.4 14,259.4	10,642.0 10,763.0	±841.3 ±977.6	189.6 226.8	144.0 181.0	±17.3 ±16.8

OTU, operational taxonomic unit; SE, standard error.

## Data Availability

The data presented in this study are available on request from the corresponding author.

## Supplementary Materials

The following are available online at https://www.mdpi.com/article/10.3390/cancers13184687/s1, Figure S1: Alpha diversity of microbiome defined by various indices in 4 sample types, Figure S2: Changes in the relative abundances of individual phyla in gastric cancer in 4 sample types, Figure S3: Comparison of gastric cancer diagnostic prediction models between healthy controls and EGC or AGC in 4 sample types.
