# Supplementary material for: Bacteria-Derived Extracellular Vesicles in Urine as a Novel Biomarker for Gastric Cancer: Integration of Liquid Biopsy and Metagenome Analysis
Source: Cancers (Basel). 2021 Sep 18;13(18):4687. doi: 10.3390/cancers13184687 (PMC8468964; doi:10.3390/cancers13184687)
Supplement: Supplementary file 1 [file cancers-13-04687-s001.zip › cancers-1369595-supplementary.v4.pptx]

## Slide 1
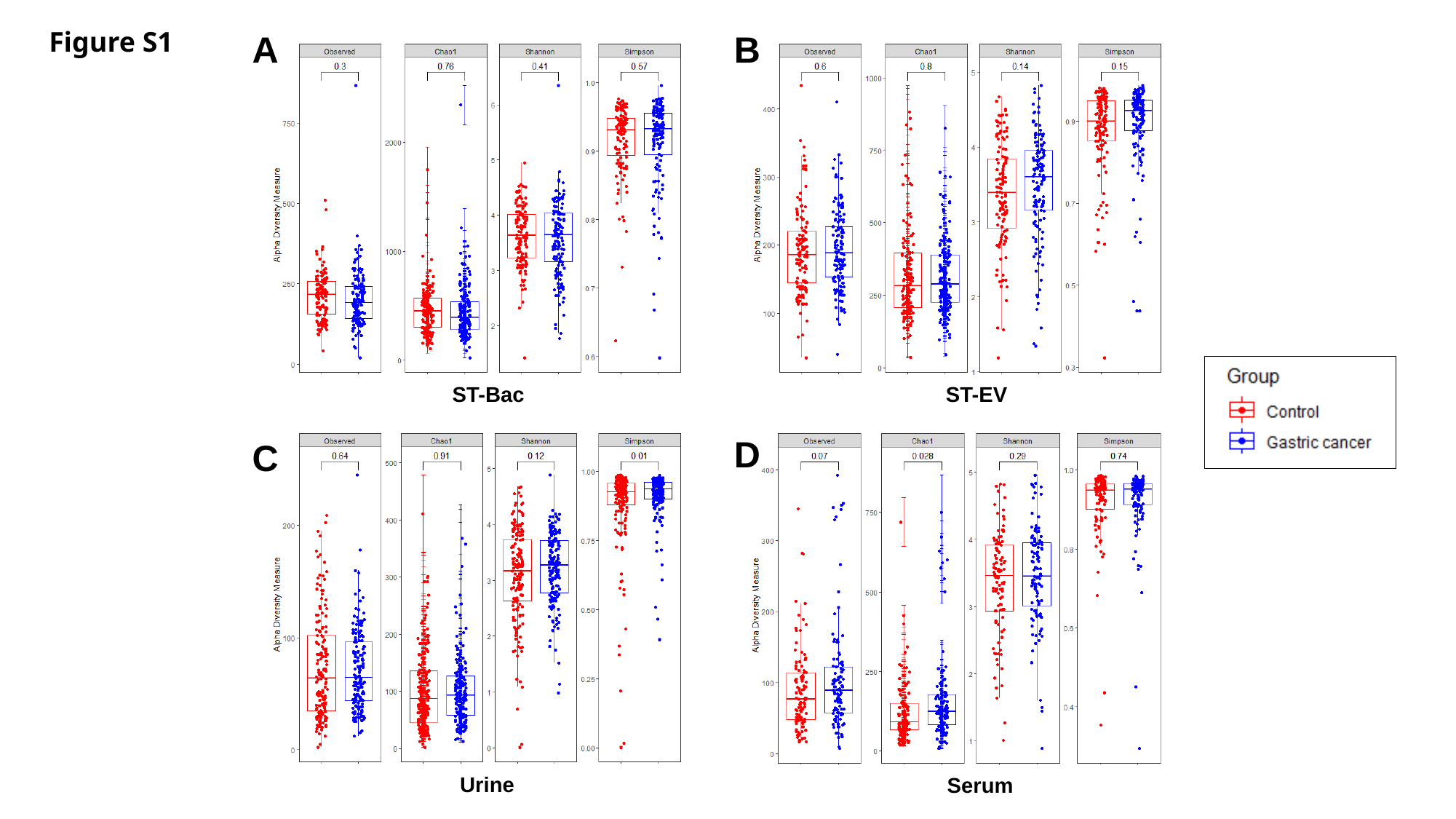

Figure S1
B
A
ST-Bac
ST-EV
D
C
Urine
Serum

## Slide 2
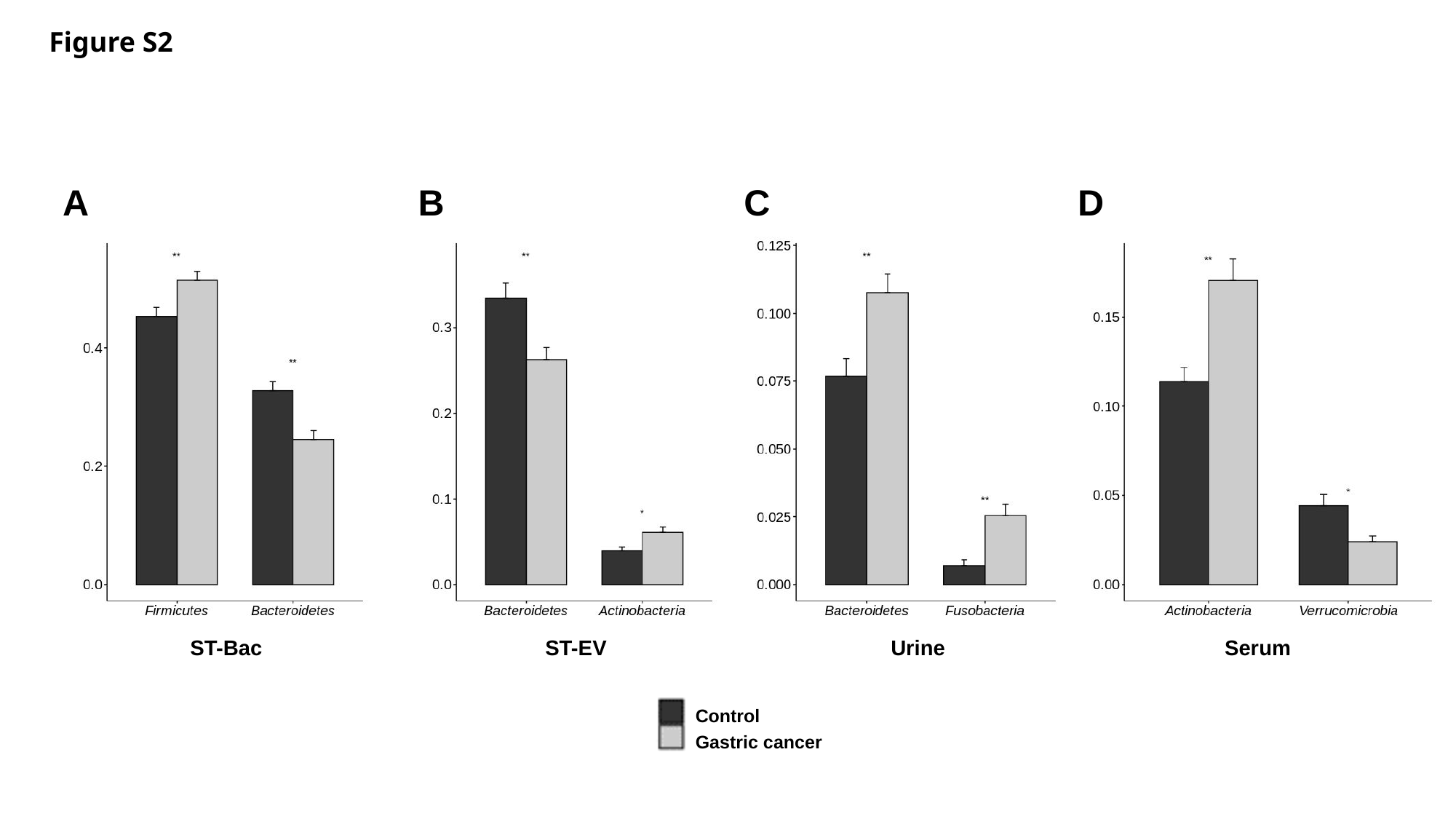

Figure S2
B
C
D
A
ST-EV
Urine
Serum
ST-Bac
Control
Gastric cancer

## Slide 3
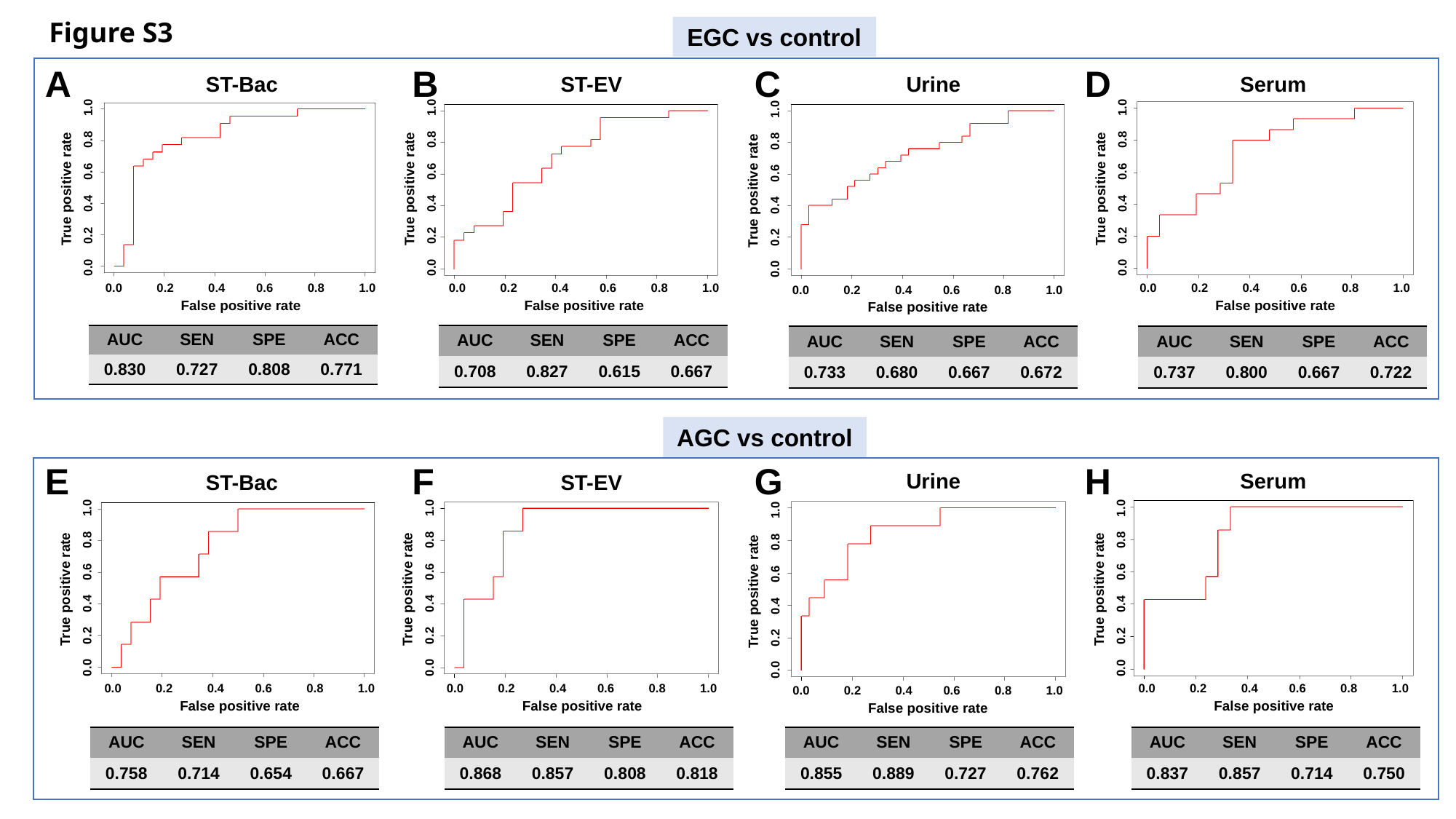

Figure S3
EGC vs control
B
C
D
A
ST-EV
Urine
Serum
ST-Bac
True positive rate
0.0 0.2 0.4 0.6 0.8 1.0
0.0 0.2 0.4 0.6 0.8 1.0
False positive rate
True positive rate
0.0 0.2 0.4 0.6 0.8 1.0
0.0 0.2 0.4 0.6 0.8 1.0
False positive rate
True positive rate
0.0 0.2 0.4 0.6 0.8 1.0
0.0 0.2 0.4 0.6 0.8 1.0
False positive rate
True positive rate
0.0 0.2 0.4 0.6 0.8 1.0
0.0 0.2 0.4 0.6 0.8 1.0
False positive rate
| AUC | SEN | SPE | ACC |
| --- | --- | --- | --- |
| 0.830 | 0.727 | 0.808 | 0.771 |
| AUC | SEN | SPE | ACC |
| --- | --- | --- | --- |
| 0.708 | 0.827 | 0.615 | 0.667 |
| AUC | SEN | SPE | ACC |
| --- | --- | --- | --- |
| 0.733 | 0.680 | 0.667 | 0.672 |
| AUC | SEN | SPE | ACC |
| --- | --- | --- | --- |
| 0.737 | 0.800 | 0.667 | 0.722 |
AGC vs control
F
G
H
E
Urine
Serum
ST-EV
ST-Bac
True positive rate
0.0 0.2 0.4 0.6 0.8 1.0
0.0 0.2 0.4 0.6 0.8 1.0
False positive rate
True positive rate
0.0 0.2 0.4 0.6 0.8 1.0
0.0 0.2 0.4 0.6 0.8 1.0
False positive rate
True positive rate
0.0 0.2 0.4 0.6 0.8 1.0
0.0 0.2 0.4 0.6 0.8 1.0
False positive rate
True positive rate
0.0 0.2 0.4 0.6 0.8 1.0
0.0 0.2 0.4 0.6 0.8 1.0
False positive rate
| AUC | SEN | SPE | ACC |
| --- | --- | --- | --- |
| 0.758 | 0.714 | 0.654 | 0.667 |
| AUC | SEN | SPE | ACC |
| --- | --- | --- | --- |
| 0.868 | 0.857 | 0.808 | 0.818 |
| AUC | SEN | SPE | ACC |
| --- | --- | --- | --- |
| 0.855 | 0.889 | 0.727 | 0.762 |
| AUC | SEN | SPE | ACC |
| --- | --- | --- | --- |
| 0.837 | 0.857 | 0.714 | 0.750 |
